# Iodine deficiency profile of Central Java province Indonesia during the year 2011

**DOI:** 10.1186/1687-9856-2013-S1-P149

**Published:** 2013-10-03

**Authors:** Saldi Fitra, Rudy Susanto, Asri Purwanti, Agustini Utari

**Affiliations:** 1 Department of Pediatrics, Faculty of Medicine, Diponegoro University / Dr. Kariadi Hospital, Semarang, Indonesia

## 

Iodine Deficiency Disorders (IDD) has recognised as the most common preventable cause of brain damage. IDD also one of the nutrition problems that influences human growth and development. In Indonesia, about 50-60% of the areas are IDD endemic areas. Prevention of IDD in 10 step should be done every year. The aims of this study was to evaluate IDD prevention program in Central Java, Indonesia.

This study was done in 15 district ( 289 sub district ) of Central Java Province during the year 2011. A total of 55617 neonate, 106825 pregnant women, and 2958 urine samples of pregnant women were examined. Neonatal Hypothyroid Index (NHI) in the first week of neonatal visit, Total Goiter Rate (TGR), Urine Iodine Excretion (UIE) of pregnant women and household consumption of iodized salt were collected. The blood analysis was done in IDD Laboratory in Bandung and UIE analysis was done in BP GAKY Magelang, Indonesia.

Of 55617 neonate, there were 18 neonatus (0,03%) suspected as cretins. TGR degree 1-2 was found in 174 cases (0,18%). The median of pregnant women UIE was 156mg/l and UEI <100mg/l was found in 1002 (33,87%) pregnant women. There were 4 district with mild iodine deficiency. The consumption of iodized salt in the population was 81,6%.

In the future, the 33,87% of pregnant women with UIE <100 mg/l will put 1/3 of babies born in these area in the risk of brain damage. Problem of iodine deficiency in the past with TGR 0,18 % and 18 neonates with suspected cretins is iceberg phenomena with more brain damage. Screening for congenital hypothyroidism should be performed every year in the newborn, followed by UIE examination of childbearing age women and pregnant women because it can happen iodine replate area and new endemic areas.

